# Peak systolic velocity ratio derived from quantitative vessel analysis for restenosis after femoropopliteal intervention: a multidisciplinary review from Endovascular Asia

**DOI:** 10.1007/s12928-019-00602-z

**Published:** 2019-07-11

**Authors:** Osami Kawarada, Koji Hozawa, Kan Zen, Hsuan-Li Huang, Su Hong Kim, Donghoon Choi, Kihyuk Park, Kenichi Kato, Taku Kato, Yoshinori Tsubakimoto, Shigeo Ichihashi, Naoki Fujimura, Akihiro Higashimori, Tomoyasu Sato, Bryan Ping-Yen Yan, Skyi Yin-Chun Pang, Chumpol Wongwanit, Yew Pung Leong, Benjamin Chua, Robbie K. George, I-Chih Chen, Jen-Kuang Lee, Chung-Ho Hsu, Uei Pua, Yo Iwata, Kojiro Miki, Kozo Okada, Hideaki Obara

**Affiliations:** 1 Department of Cardiovascular Medicine, Ikuwakai Memorial Hospital, 3-20-29 Tatsumikita, Ikunoku, Osaka, Osaka 544-0004 Japan; 2 Department of Cardiology, New Tokyo Hospital, Matsudo, Japan; 3 Department of Cardiovascular Medicine, Kyoto Prefectural University of Medicine, Kyoto, Japan; 4 Division of Cardiology, Taipei Tzu Chi Hospital, Buddhist Tzu Chi Medical Foundation, New Taipei, Taiwan; 5 Department of Cardiology, Busan Veterans Hospital, Busan, Korea; 6 Division of Cardiology, Severance Cardiovascular Hospital, Yonsei University College of Medicine, Seoul, Korea; 7 Department of Vascular Surgery, Daegu Catholic University Hospital, Daegu, Korea; 8 Department of Vascular Laboratory, Ikuwakai Memorial Hospital, Osaka, Japan; 9 Department of Cardiology, Rakuwakai Otowa Hospital, Kyoto, Japan; 10 Department of Cardiology, Kyoto Second Red Cross Hospital, Kyoto, Japan; 11 Department of Radiology, Nara Medical University, Kashihara, Japan; 12 Division of Vascular Surgery, Tokyo Saiseikai Central Hospital, Tokyo, Japan; 13 Department of Cardiology, Kishiwada Tokushukai Hospital, Kishiwada, Japan; 14 Department of Radiology, Tsuchiya General Hospital, Hiroshima, Japan; 15 Department of Medicine and Therapeutics, Prince of Wales Hospital, The Chinese University of Hong Kong, Sha Tin, Hong Kong China; 16 Department of Surgery, Pamela Youde Nethersole Eastern Hospital, Chai Wan, Hong Kong China; 17 Department of Vascular Surgery, Siriraj Hospital, ‎Bangkok, Thailand; 18 Department of Vascular Surgery, Cardiac Vascular Sentral Kuala Lumpur, Kuala Lumpur, Malaysia; 19 Department of Vascular Surgery, Vascular and Interventional Centre Singapore, Mount Elizabeth Novena Specialist Centre, The Farrer Park Hospital, Singapore, Singapore; 20 Department of Vascular Surgery, Narayana Hrudayalaya and Mazumdar Shaw Medical Centre, Bengaluru, India; 21 Division of Cardiology, Department of Internal Medicine, Tainan Municipal Hospital, Tainan, Taiwan; 22 Department of Cardiology, National Taiwan University, Taipei, Taiwan; 23 Department of Cardiology, China Medical University Hospital, Taichung, Taiwan; 24 Department of Radiology, Tan Tock Seng Hospital, Singapore, Singapore; 25 Department of Cardiology, Funabashi Municipal Medical Center, Funabashi, Japan; 26 Department of Cardiology, Hyogo College of Medicine, Nishinomiya, Japan; 27 Department of Cardiology, Yokohama City University Medical Center, Yokohama, Japan; 28 Department of Surgery, Keio University School of Medicine, Tokyo, Japan

**Keywords:** Methodology, Angiography, Ultrasound, Restenosis, Intervention

## Abstract

With technological improvements in the endovascular armamentarium, there have been tremendous advances in catheter-based femoropopliteal artery intervention during the last decade. However, standardization of the methodology for assessing outcomes has been underappreciated, and unvalidated peak systolic velocity ratios (PSVRs) of 2.0, 2.4, and 2.5 on duplex ultrasonography have been arbitrarily but routinely used for assessing restenosis. Quantitative vessel analysis (QVA) is a widely accepted method to identify restenosis in a broad spectrum of cardiovascular interventions, and PSVR needs to be validated by QVA. This multidisciplinary review is intended to disseminate the importance of QVA and a validated PSVR based on QVA for binary restenosis in contemporary femoropopliteal intervention.

## Introduction

The burden of atherosclerotic peripheral artery disease (PAD) is projected to increase globally [1]. The femoropopliteal (FP) artery is the most common site of PAD involvement. With technological improvements in the endovascular armamentarium, catheter-based FP intervention has gained popularity during the last decade [2–7]. Although the need for quantitative vessel analysis (QVA) for objective evaluation in FP intervention outcomes has been emphasized for over a decade [8], a standard methodology for assessing restenosis has yet to be established.

Meanwhile, because of its noninvasive nature, repeatability, and lack of a need for contrast agents, duplex ultrasonography (DUS) has been widely used without scientific validation in the identification of restenosis after FP intervention. In order to correct this chaotic situation, there is a strong movement to investigate the relationship between the peak systolic velocity ratio (PSVR) based on DUS and restenosis based on QVA [9, 10]. This multidisciplinary review from Endovascular Asia is intended to disseminate the importance of QVA and a validated PSVR based on QVA for binary restenosis in contemporary FP intervention.

## QVA in FP intervention

### Need for dissemination of QVA

Visual interpretation of angiography is subject to substantial intra- and inter-observer variability. Therefore, the methodology of QVA was initially introduced as quantitative coronary analysis (QCA) in the field of coronary intervention in the mid-1980s to permit more objective, accurate, and reproducible visual assessment of lesion severity compared to angiography [11]. QCA has been developed not only to qualify lesion severity, but also to objectively evaluate the outcomes of endovascular therapy, including balloon angioplasty or stent. Thanks to the dissemination of this standard methodology, QCA has offered highly insightful findings as a result of landmark clinical trials and daily clinical practice [12]. Given the history of coronary intervention, an awareness of the importance of QVA is required for the development of FP intervention.

### Methodology of FP QVA

**Fig. 1:**
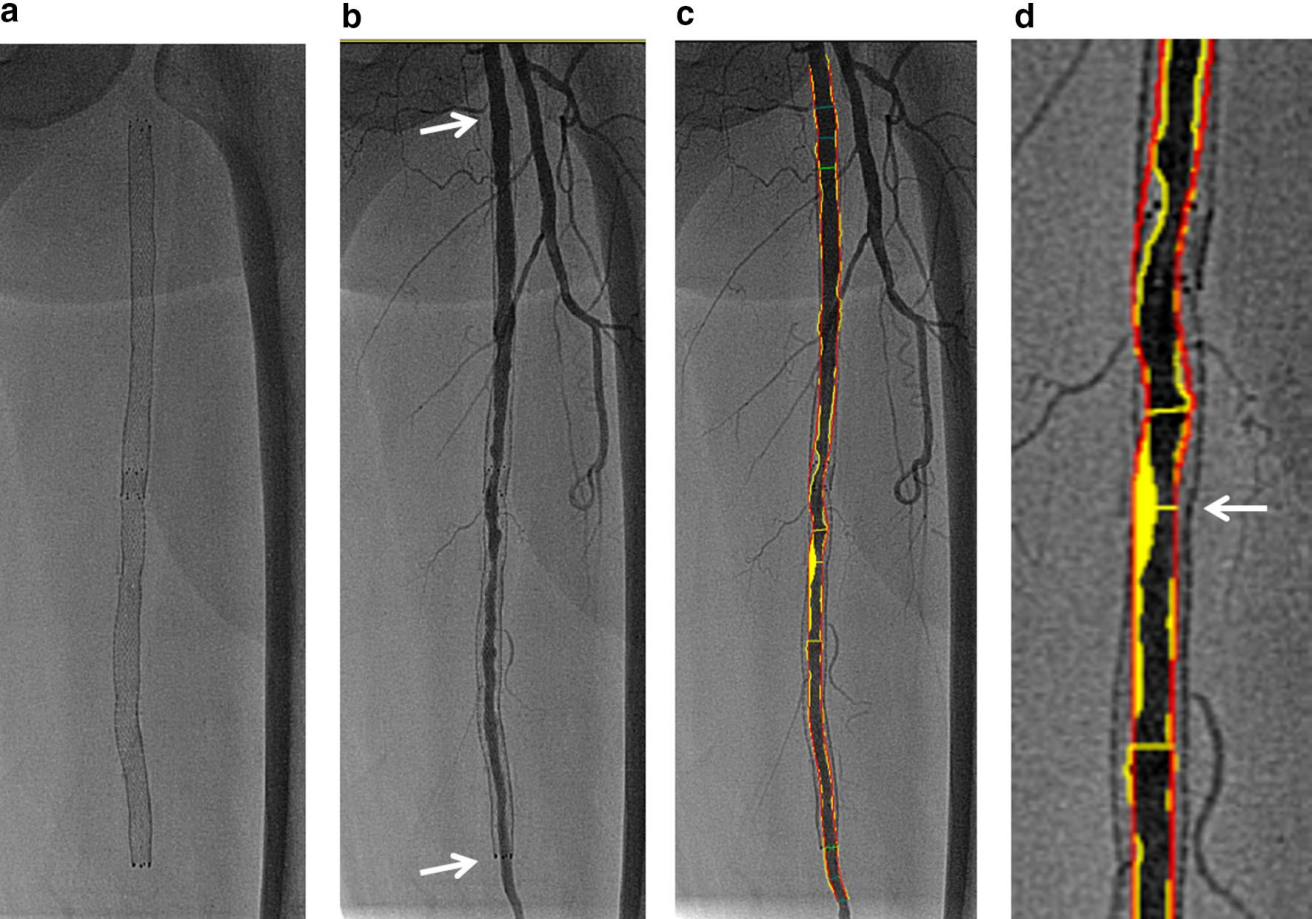
Quantitative FP artery analysis (citation from Ref. [10]). **a** Nitinol stents implanted in the left FP artery. **b** Angiography showing intimal hyperplasia in the stented femoropopliteal artery. Note the proximal and distal edges of the nitinol stent (arrows). **c** Automatically applied tracings show lumen contour (yellow lines) and assumed vessel (red lines). **d** Magnified view of the minimum lumen diameter within the stent (arrow). The minimum lumen diameter can be determined based on the lumen contour (yellow lines), and the assumed vessel (red lines) can be used as the reference vessel

**Fig. 2:**
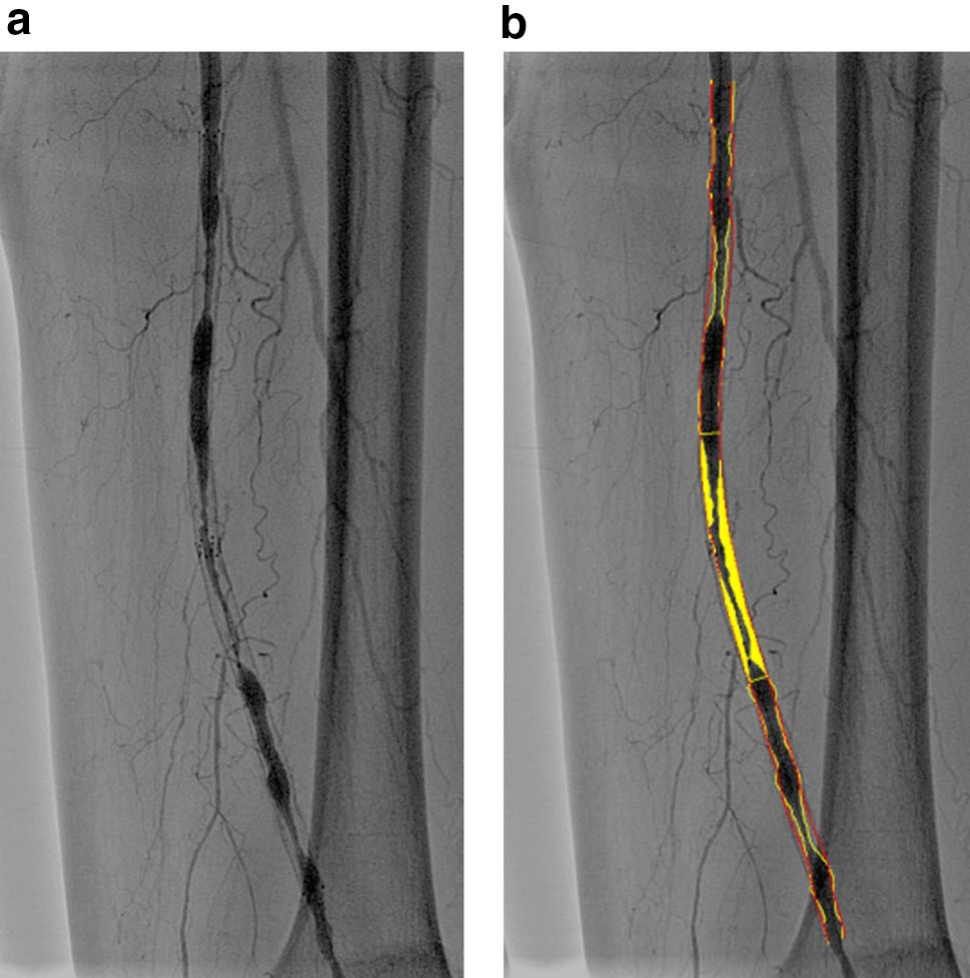
QVA for multiple restenosis. **a** Angiography showing multiple stenoses due to intimal hyperplasia in a stent in the mid-distal FP artery. **b** In the QVA, the most critical restenosis can be depicted by automatically applied tracings of lumen contour (yellow lines) and assumed vessel (red lines). The % diameter stenosis is 76.9%, suggesting binary restenosis

**Fig. 3:**
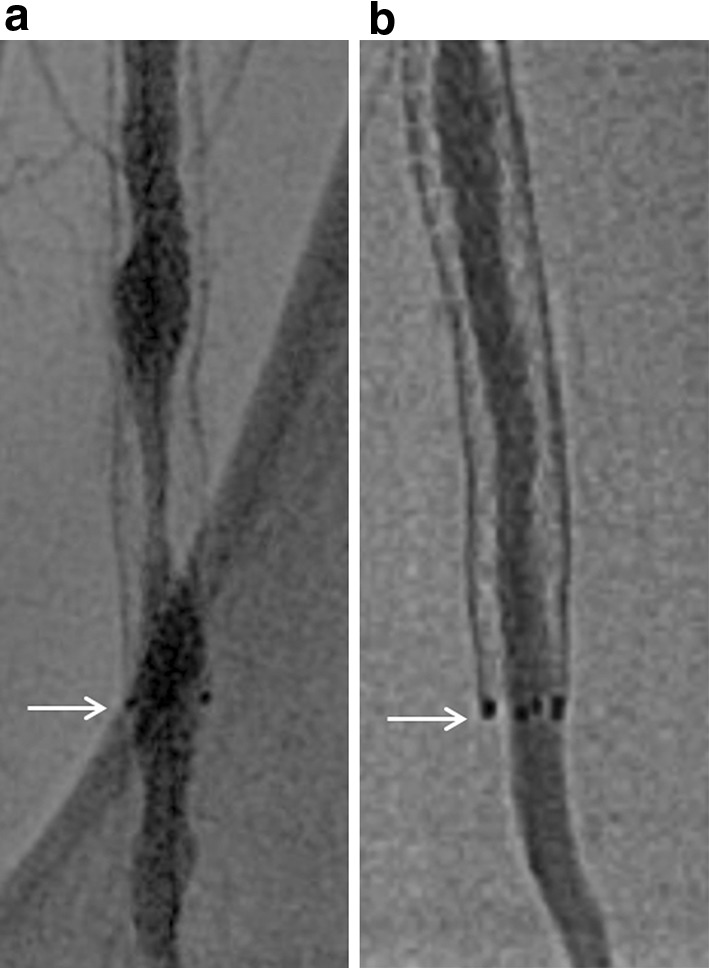
Difference in stent edge immediately after nitinol stenting and in follow-up angiography. There are 2 types of angiographic appearance at the stent edge in follow-up angiography. **a** Type A. No gap between the stent line and the intraluminal line outside the stent (arrow), **b** Type B. A gap caused by significant intimal hyperplasia and stent expansion at the stent edge between the stent line and the intraluminal line outside the stent (arrow)

For QVA, an angiogram of the entire FP artery needs to be obtained using the anteroposterior and/or oblique view. To determine lesion severity, QVA using an automated edge detection algorithm should be performed in a blinded fashion (Fig. 1). In most cases, a catheter tip placed at the common femoral artery is unavailable as a calibration method because movement of the catheterization table is required for angiographic evaluation of the entirety of FP lesions. It is therefore impossible to calculate the reference vessel and lumen diameters and lesion length, and only the percent diameter stenosis (%DS) can be calculated as an indicator of the degree of restenosis based on a lumen contour and an assumed vessel as the reference vessel that are automatically drawn in the QVA system. The formula of %DS is as follows; minimum lumen diameter in the lesion or within the stent/the assumed vessel diameter. If the entire FP artery is stented, then the control segment, which is evaluated and measured against in-stent stenosis, is defined as being within a widely patent segment of the proximal or distal stent. In cases of multiple restenosis, the most critical restenosis can be identified (Fig. 2). Also, in cases of nitinol stenting with continued radial force and expansion over time, greater attention might need to be paid at follow-up to the gap elicited between the stent line and the luminal edge (Fig. 3). Fifty percent diameter stenosis is considered to be significant based on theoretical and experimental studies [13]. Indeed, angiographically detected lesions with a %DS of 50% or greater have been historically considered to be a dichotomous event, or “binary restenosis”, in the field of cardiovascular intervention [14]. Thus, binary restenosis is defined as a %DS > 50% on QVA for each lesion or within the stent.

## PSVR in FP intervention

### DUS as an alternative to angiography

Over 3 decades ago, Jager et al. [15] proposed a system for classifying the degree of arterial stenosis in the lower limbs on the basis of the Doppler waveform shape, the degree of spectral broadening, and the increase in peak systolic velocity (PSV) within the stenosis. However, spectral broadening was too subjective and the waveform shape was affected by a variety of factors, including cardiac output and rhythm, resistance of the vascular bed, integrity of the intima, and both proximal and distal disease [16–18]. PSVR was found to be more closely correlated with the degree of stenosis than PSV [19]. This was the beginning of PSVR as an alternative clinical index to angiographic narrowing.

### Practice of DUS

**Fig. 4:**
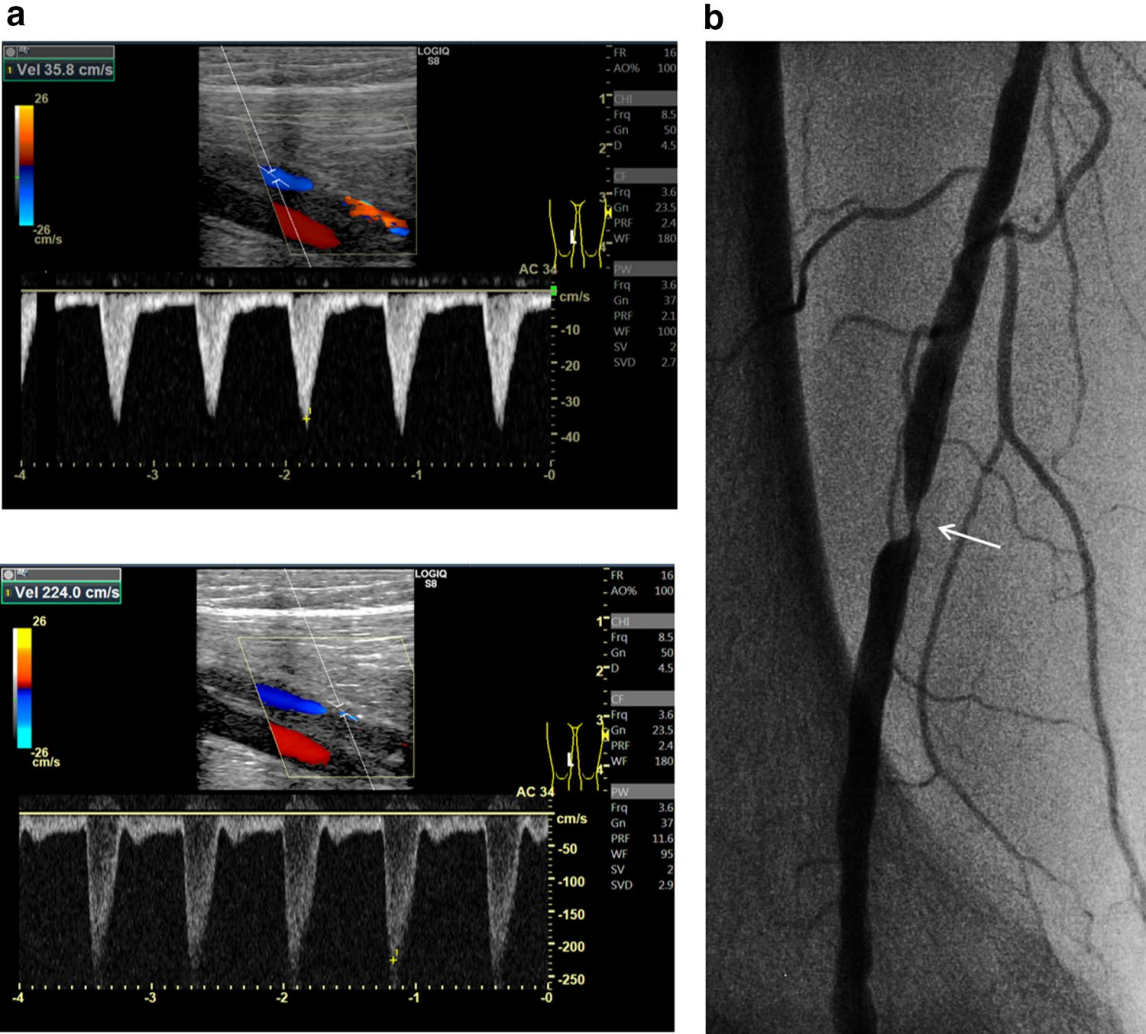
DUS for restenosis after balloon angioplasty. **a** The proximal PSV is 35.8 cm/s (upper) and the PSV at the point of stenosis is 224.0 cm/s (lower). The PSVR is 6.26, suggesting binary restenosis. **b** In accordance with DUS findings, confirmatory angiography shows restenosis in the distal FP artery (arrow)

**Fig. 5:**
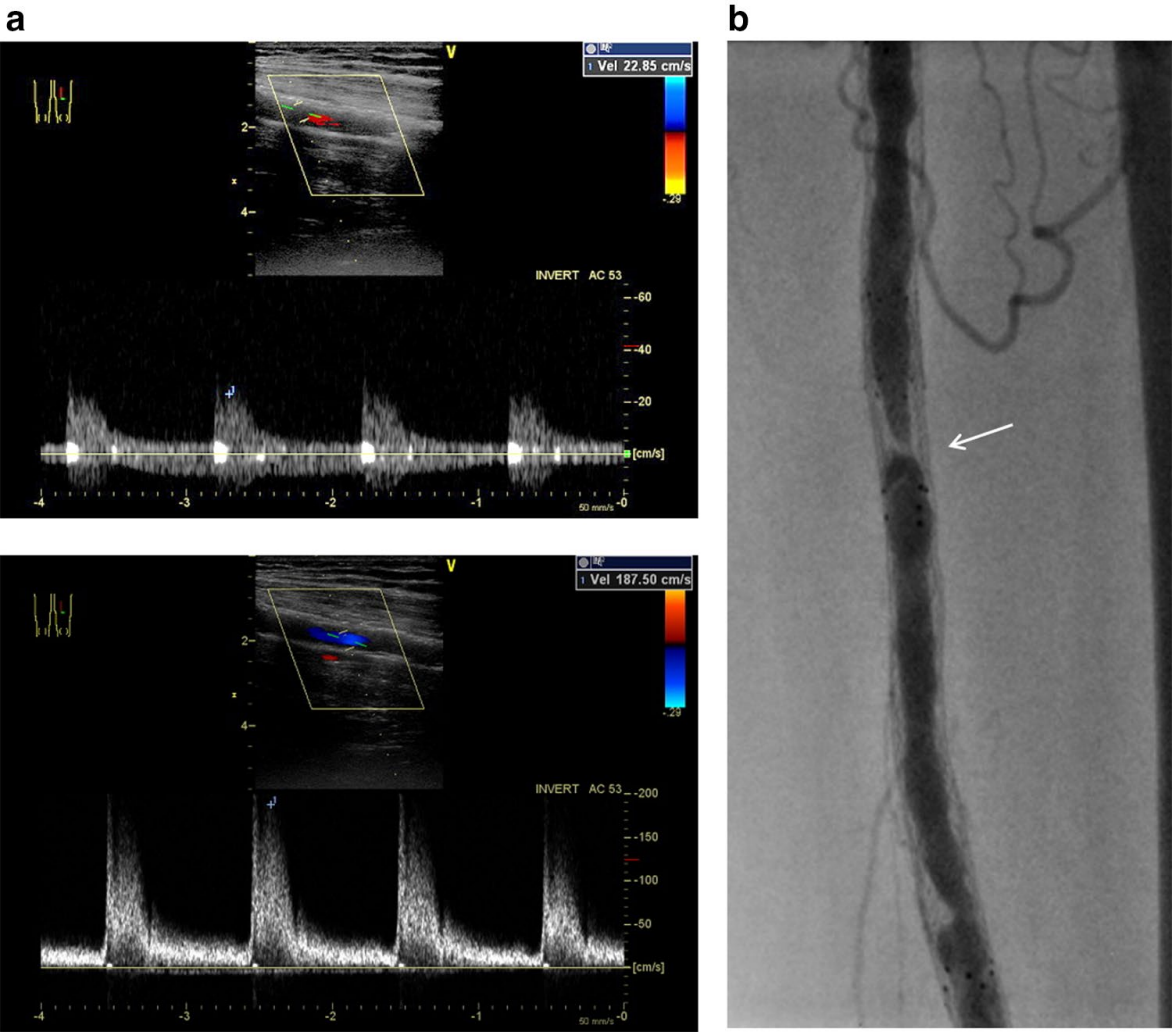
DUS for in-stent restenosis after nitinol stenting. **a** The proximal PSV is 22.9 cm/s (upper) and the PSV at the point of stenosis is 187.5 cm/s (lower). The PSVR is 8.20, suggesting binary in-stent restenosis. **b** In accordance with DUS findings, confirmatory angiography shows in-stent restenosis in the mid-segment of the stented FP artery (arrow)

DUS employing a commercially available machine should be performed by experienced vascular specialists. All patients are examined in a supine position using a duplex scanner with a 7.5- or 8-MHz transducer [9, 10]. The segment of interest after balloon angioplasty or stenting can be visualized using combined B-mode and color-Doppler ultrasound. The Doppler signal is acquired at an angle of 60 degrees or as small as possible, and velocity spectra are recorded proximal to and at the site of maximum flow disturbance. Doppler spectral analysis can determine the highest PSV (PSV at the lesion) as well as the PSV in the area adjacent to the normal-looking segment (PSV proximal). PSVR can be calculated by the following formula: PSV at the lesion/PSV proximal (Figs. 4, 5).

### Less-validated PSVR without the use of QVA

In the 1990s, the relationship between DUS and angiography was evaluated, and it was suggested that on angiography, 50% diameter reduction by inherently flawed “visual estimation” was equivalent to a PSVR of 2.0–3.0 in the lower limb arteries, including the femoral artery, while it was possible to have different PSVR cut-off points for the iliac, common femoral, superficial femoral, popliteal, and crural arteries [19–23]. Even in the 2000s, two studies that did not involve the use of QVA focused on the optimal PSVR in the FP artery (Table 1) [24, 25]. According to the study of Schlager et al. [24], in which the majority of cases were de novo lesions (de novo lesion in 97%, restenosis in 3%), a PSVR of 2.4 indicated 50% stenosis with a sensitivity of 81%, specificity of 93%, positive predictive value (PPV) of 84%, and negative predictive value (NPV) of 91%. According to the study of Baril et al. [25], PSVR was more accurate than PSV, and a PSVR of 1.5 yielded a sensitivity of 93%, specificity of 89%, PPV of 96%, and NPV of 81% in terms of estimating in-stent restenosis. Reflecting the lack of scientifically validated PSVR values, PSVRs of 2.0, 2.4, and 2.5 have been arbitrarily but routinely employed, even in contemporary FP intervention studies. Therefore, scientifically validated PSVR criteria based on QVA are indispensable.

**Table 1:** PSVR for 50% de novo stenosis or restenosis in the femoropopliteal artery

References	Method of angiography analysis	Native/stented artery	Denovo/restenosis lesion	PSVR criteria for 50% stenosis	Sens. (%)	Spec. (%)	PPV (%)	NPV (%)
Polak et al. [20]	Visual estimation	Native	Denovo	2	88	95	NR	NR
Legemate et al. [21]	Visual estimation	Native	Denovo	2.5	65	97	69	96
Leng et al. [19]	Visual estimation	Native	Denovo	3	70	96	95	74
Aly et al. [23]	Visual estimation	Native	Denovo	2	95	99	94	99
Schlager et al. [24]	Visual estimation	Native (97%) and stented (3%)	De novo and restenosis	2.4	81	93	84	91
Baril et al. [25]	Visual estimation	Stented	Restenosis	1.5	93	89	96	81
Kawarada et al. [10]	Quantitative vessel analysis	Stented	Restenosis	2.85	88	84	85	88
Macharzina et al. [9]	Quantitative vessel analysis	Native	Restenosis (single)	2.6	98	94	98	94
			Restenosis (multisegmental)	2.6	87	93	45	99

*PSVR* peak systolic velocity ratio, *Sens* sensitivity, *Spec* specificity, *PPV* positive predictive value, *NPV* negative predictive value, *NR* not reported

### Derivation of PSVR threshold for restenosis based on QVA

In the 2010s, 2 retrospective studies (the study of Kawarada et al. in 2013 and that of Macharzina et al. in 2015) investigated the relationship between DUS parameters and %DS derived by QVA in the context of restenosis after bare-metal nitinol stenting and balloon angioplasty in the FP arteries [9, 10]. In these studies, compared to PSV, PSVR yielded a better correlation with %DS, suggesting that PSVR can provide better performance than PSV in terms of correlation with angiographic narrowing, both in unstented and stented FP lesion assessment.

**Fig. 6:**
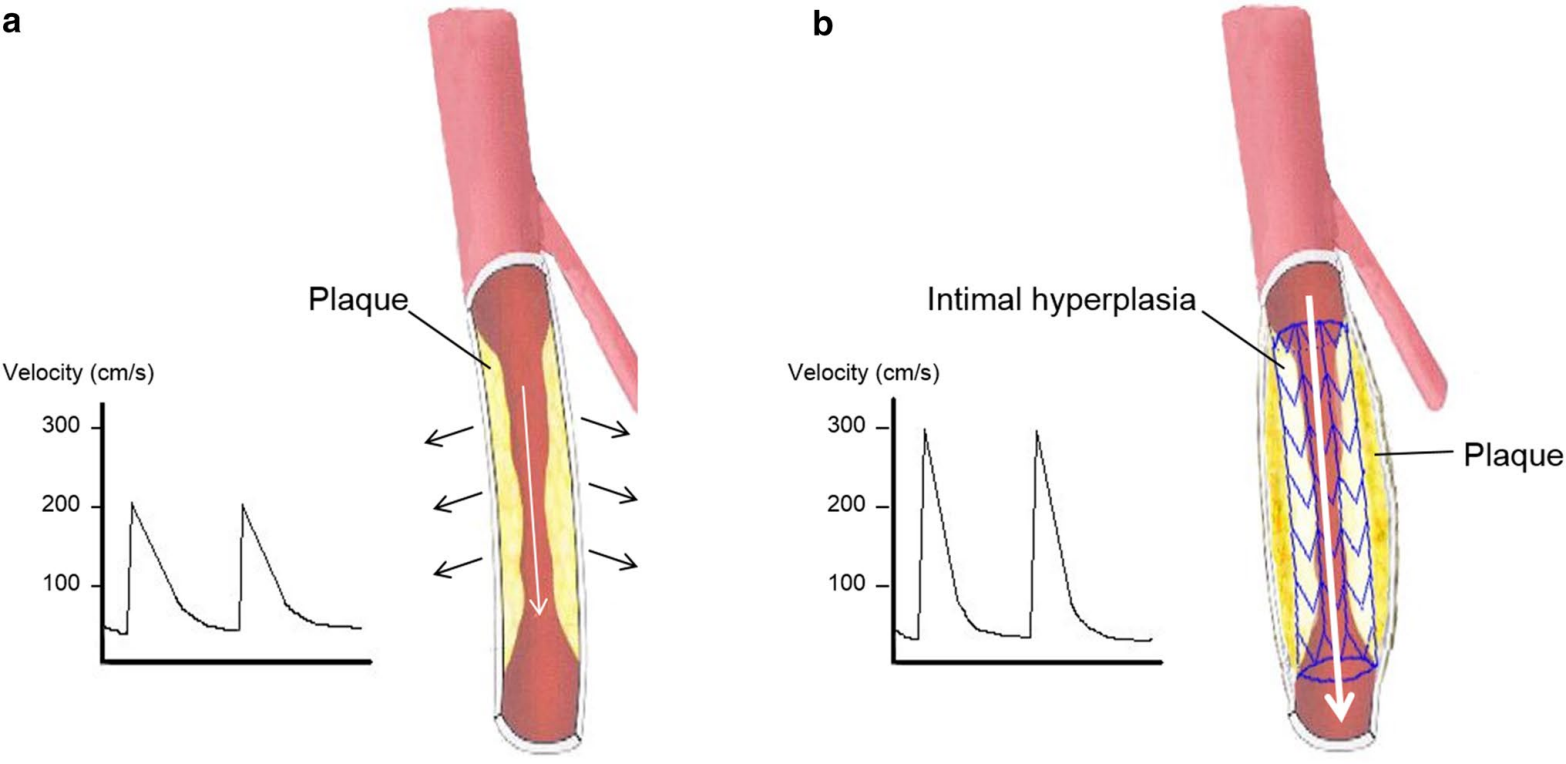
Illustration showing differences between an unstented and stented FP artery in terms of the effect of vessel compliance on flow velocity (citation from Ref. [10]). **a** Unstented FP artery. **b** Stented FP artery. The stented FP artery has less compliance than the unstented SFA. The PSV and the PSVR increased more in the stented FP artery than in the unstented FP artery, despite the same degree of stenosis

According to receiver operating characteristic (ROC) analysis in the study of Macharzina et al. [9], the optimal threshold for detecting binary restenosis in an unstented FP artery was 2.6 for a single stenosis, with a sensitivity of 98%, specificity of 94%, PPV of 98%, and NPV of 94%, compared to 2.6 for multisegmental stenoses, with a sensitivity of 87%, specificity of 93%, PPV of 45%, and NPV of 99%. These data suggest that the accuracy for multisegmental restenosis is inferior to that for single restenosis even though the optimal cut-off threshold is the same. In the study of Kawarada et al. [10], ROC analysis identified a PSVR of 2.85 as the best cut-off criterion for restenosis in a stented FP artery, with a sensitivity of 88%, specificity of 84%, PPV of 85%, and NPV of 88% (Table 1). These findings suggest that we might need to consider a different optimal PSVR for QVA-based restenosis depending on whether the FP arteries are unstented or stented. The PSVR discrepancy between the studies of Macharzina et al. and Kawarada et al. (PSVR 2.6 and 2.85, respectively) may be due to altered arterial biomechanical properties following stent implantation, with the resultant stent–arterial complex decreasing FP artery compliance; this would in turn cause elevated blood flow velocity, PSV, and PSVR (Fig. 6).

**Fig. 7:**
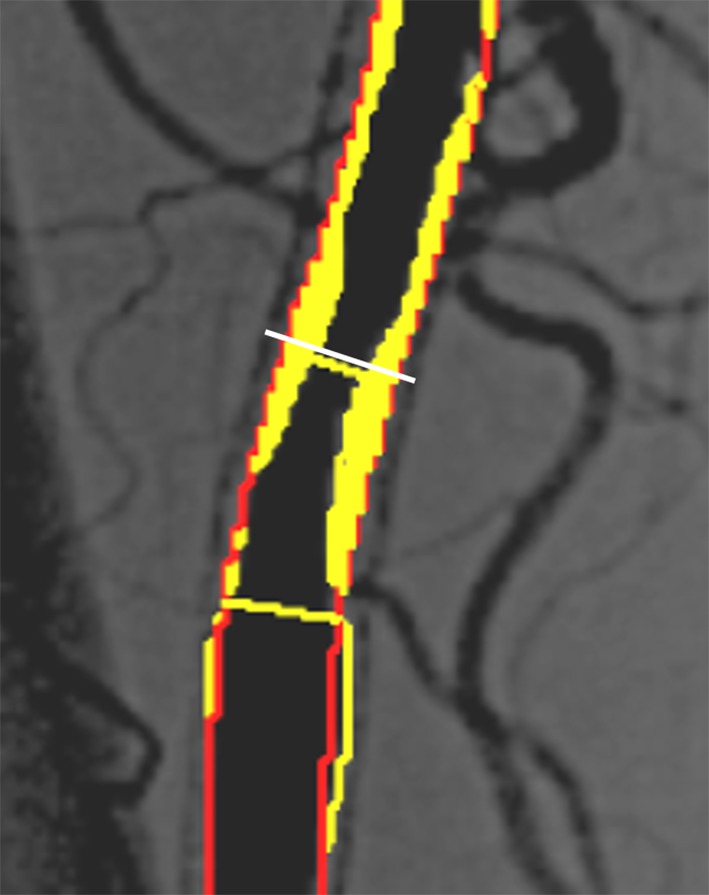
Representative sample of difference in %DS between QVA and visual estimation analysis (citation from Ref. [10]). QVA results in a %DS of 48%; however, when stent diameter is used as the reference diameter (white line), %DS based on visual estimation analysis is 65%

Furthermore, the PSVR threshold derived from QVA appears to be higher than that derived from visual estimation analysis, especially in the context of in-stent restenosis (PSVR 2.85 in Kawarada et al.’s study and PSVR 1.5 in Baril et al.'s study). In the setting of visual estimation, stent diameter can be the reference diameter, and %DS can be calculated by the following formula: (stent diameter − in-stent minimum lumen diameter)/stent diameter × 100. Therefore, %DS based on QVA could be lower than %DS based on visual estimation analysis (Fig. 7), and consequently the optimal PSVR for in-stent restenosis (50% stenosis) could be higher in QVA than in visual estimation analysis.

### Drawbacks of PSVR

**Fig. 8:**
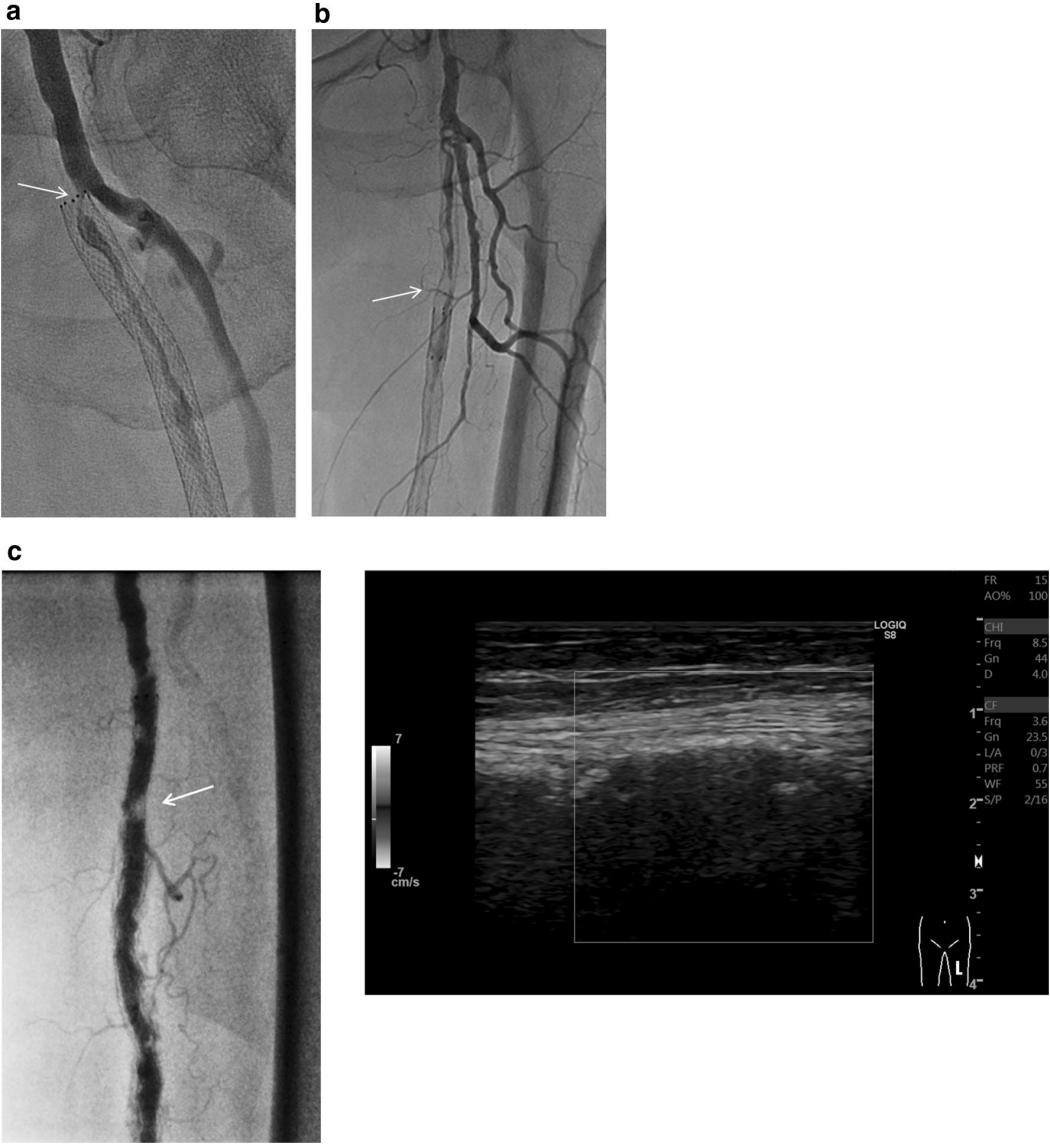
Representative cases of drawbacks of DUS for assessing restenosis. **a** Subtotal occlusion. In this stented case, there is no acceleration or increase in blood flow because of subtotal occlusion (arrow), although a Doppler color signal is present. **b** Diffuse lesion. In this stented case with critical restenosis embedded in diffuse intimal hyperplasia (arrow), determination of the proximal reference point for Doppler sample volume might be confusing. **c** Calcified lesion. In this stented case, angiography demonstrates significant in-stent restenosis (arrow) in the mid-FP artery (left). However, the underlying calcification in the arterial wall prevents visualization of the artery and measurement of velocity on DUS because of the acoustic shadow (right)

The fact that the assessment of DUS might be inconclusive in nearly 20% of cases in clinical practice needs to be taken seriously [26]. The limitations of DUS are as follows (Fig. 8): (1) subtotal reocclusion, whether stented or unstented, does not necessarily represent high-velocity flow; (2) the performance of DUS for detecting restenosis within the diffusely extended significant intimal hyperplasia is not elucidated yet; (3) during surveillance, identification of unstented lesions such as those after plain or drug-coated balloon angioplasty might be challenging due to lack of landmarks; (4) severely calcified vessels do not permit clear visualization or measurement of flow velocity; and (5) ultrasound quality is dependent on operator skill and ultrasound machine.

Unlike QVA, PSVR is potentially subject to intra- and inter-observer variability [27]. Also, it is noteworthy that flow velocity can be influenced by vessel compliance. Therefore, in addition to the extent of underlying arterial calcification and calcified intimal hyperplasia, a variety of nitinol stents with distinct mechanical properties, rigidities, degrees of continued expansion due to radial force over time, and stent platforms might potentially yield heterogenous flow velocities, PSVs, and cut-off thresholds of PSVR for restenosis. In particular, at the stent edge where vessel compliance can change drastically, PSVR might be inconsistent. Furthermore, in parallel with the development of new devices, it remains unclear whether downstream effect of drug particles or excipient from drug-eluting devices can affect flow velocity [28], and a late lumen loss after the use of drug-eluting device might be an ongoing dynamic process. We emphasize the need for recognizing the drawbacks of PSVR when applying and interpreting DUS in clinical trials and clinical practice.

## Conclusions

From the viewpoint of methodology standardization, QVA should be the mainstay in contemporary FP intervention. If PSVR is used to assess restenosis as an alternative to QVA, an optimal PSVR criteria should be derived from QVA. As of today, based on the currently available studies utilizing DUS and QVA, a PSVR of 2.6 for unstented lesions and a PSVR of 2.85 for stented lesions are valid to identify restenosis in the FP artery.
